# Modeling the impacts of climate change and technical progress on the wheat yield in inland China: An autoregressive distributed lag approach

**DOI:** 10.1371/journal.pone.0184474

**Published:** 2017-09-26

**Authors:** Shiyan Zhai, Genxin Song, Yaochen Qin, Xinyue Ye, Jay Lee

**Affiliations:** 1 College of Environment and Planning/Laboratory of Geospatial Technology for the Middle and Lower Yellow River Regions, Henan Collaborative Innovation Center for the Yellow River Civilization Heritage and Modern Civilization Construction, Henan University, Kaifeng, China; 2 Key Laboratory of Geographic Information Science, Ministry of Education, East China Normal University, Shanghai, China; 3 Department of Geography, Kent State University, Kent, Ohio, United States of America; Henan Agricultural University, CHINA

## Abstract

This study aims to evaluate the impacts of climate change and technical progress on the wheat yield per unit area from 1970 to 2014 in Henan, the largest agricultural province in China, using an autoregressive distributed lag approach. The bounded F-test for cointegration among the model variables yielded evidence of a long-run relationship among climate change, technical progress, and the wheat yield per unit area. In the long run, agricultural machinery and fertilizer use both had significantly positive impacts on the per unit area wheat yield. A 1% increase in the aggregate quantity of fertilizer use increased the wheat yield by 0.19%. Additionally, a 1% increase in machine use increased the wheat yield by 0.21%. In contrast, precipitation during the wheat growth period (from emergence to maturity, consisting of the period from last October to June) led to a decrease in the wheat yield per unit area. In the short run, the coefficient of the aggregate quantity of fertilizer used was negative. Land size had a significantly positive impact on the per unit area wheat yield in the short run. There was no significant short-run or long-run impact of temperature on the wheat yield per unit area in Henan Province. The results of our analysis suggest that climate change had a weak impact on the wheat yield, while technical progress played an important role in increasing the wheat yield per unit area. The results of this study have implications for national and local agriculture policies under climate change. To design well-targeted agriculture adaptation policies for the future and to reduce the adverse effects of climate change on the wheat yield, climate change and technical progress factors should be considered simultaneously. In addition, adaptive measures associated with technical progress should be given more attention.

## Introduction

Agriculture is one of the systems that are most susceptible to climate change because meteorological variables often dictate resource availability and control the fundamental processes involved in crop growth and development [1]. Many studies have shown that climate change has negatively or positively impacted the wheat yield in regions of high wheat yield worldwide [2–13]; however, these impacts are uncertain, and their spatial pattern, severity and driving mechanisms are unknown [14–16]. China is the world’s most important wheat yield and consumption base; from 2009 to 2014, the average yield was over 120 Mt [17]. It has been predicted that climate change poses a serious threat to China’s food security. Such a threat has been projected to escalate from 2020 to 2050 [15].

Research on the impact of climate change on wheat growth and yield has been a hot topic in China [3–5, 15, 18–19]. In the field of natural science, there are two approaches to estimating the potential impacts of climate change on agricultural systems: Exploring the effects of long-term climate change/variability on the wheat yield (1) using crop simulation models [20, 21] (e.g., CERES-Wheat, C-CAM, EPIC, and others) and climate change scenarios [7, 16, 22] and (2) using field experiments or artificial climate chamber experiments [23]. Crop simulation modeling is the most commonly used approach and is often used in combination with climate change scenarios and crop simulation models. However, modeling typically relies on many input factors, such as rainfall, soil, temperature, nutrition, evapotranspiration, the economic environment, atmospheric circulation, and carbon circulation, which make the approach laborious and expensive. In addition, uncertainties in parameter values substantially increase the uncertainty estimates for model projections [6]. And researchers do not fully understand the mechanisms driving crop growth; this lack of understanding leads to prediction risk. Field experiments require more time and a larger budget than other approaches, but the results of field experiments are often more straightforward [24].

By contrast, in the field of socioeconomics, which is based on statistical data, researchers study impacts using empirical models (e.g., panel data models, regression models) [18, 25–27] and economic models (e.g., yield function, Ricardian models) [28–29]. The main advantages of these statistical models are their transparent assessment of the model uncertainties and their decreased reliance on field calibration data; thus, these types of models are regarded as a common alternative to process-based models [6]. In addition, the related literature indicates that progress in agricultural technology has been the main driving force promoting increased wheat yield in major wheat-producing regions of the world over the past three decades [28–31]. Especially since 1978, the development of China’s agricultural biochemistry technology, mechanical technology, and cultivation technology has been rapid; in addition, the use of chemical fertilizers and large agricultural machinery (such as cutters, drills, rotary cultivators) and the construction of irrigation and water conservancy facilities have also played an important role in increasing wheat yield [28, 32–34].

Although there has been considerable research on the response mechanisms of crop growth and yield to climate change using crop models and climate change scenarios [21, 35] and using statistical approaches based on historical data from experimental stations [10, 26] or on the impacts of progress in agricultural technology [31, 36], there has been surprisingly little empirical quantitative analysis concerning the effects of climate change and progress in agricultural technology on crop productivity. In particular, there are uncertainties regarding the impacts of climate change and agricultural technology progress on wheat yield and its magnitude, the driving mechanisms and the spatial patterns of these factors [14, 37].

Henan Province is located between the central and eastern regions of China, and it represents a climatic transition zone from the subtropical zone to the warm temperate zone [5]. The region has sufficient precipitation and heat resources to support wheat cultivation. Additionally, Henan Province is a major grain-producing area that provides more than 10% of China’s food supplies. The province is therefore very important for China’s national food security. According to the National Bureau of Statistics of China (2014), wheat yields have steadily increased in Henan Province from 868 million tons in 1978 to 3,177 million tons in 2013[38].

In order to duplicate such growth in other regions and provide a reference for similar issues in other regions, to improve predictions of effects of the climate change and agricultural technology progress and to design well-targeted agriculture adaptation policies for the future, it may be desirable to identify the causal drivers of the increase in wheat yields, whether they be climate change or technological progress (e.g., fertilizer use, agricultural machinery use).

The autoregressive distributed lag (ARDL) bound test as discussed by Pesaran et al. [39] enabled us to ascertain whether a long-run relationship among the variables existes. This approach has been used in some recent empirical studies examining the impact of climate change and other factors on agriculture in many regions, such as Europe [40], Pakistan [41], Ghana [42], and India [43]. It differs from previous methods in that this method can be used to identify long-run/short-run relationship among variables. In addition, ARDL can be applied irrespective of the order of integration of the variables and it is suitable for small data samples. Therefore, our study employed ARDL model to examine the impacts of different variables on the wheat yield.

The objective of this study is to perform an in-depth study of Henan Province that explores the diversity of responses of the wheat yield to climate change and technical progress and, furthermore, to more closely investigate this subject using the ARDL approach based on historical data.

The rest of the paper proceeds as follows: Section 2 briefly reviews the literature on the relationship between climate change, technical progress and the wheat yield. Section 3 describes the study area. Section 4 discusses the data used in the study and the analytical methods employed to analyze the data. Section 5 discusses the empirical results from testing the associations between wheat yield, climate change and technical progress. Section 6 discusses previous research results and future research. Finally, Section 7 presents our findings and discusses their policy implications.

## Literature review

It has been predicted that, due to higher atmospheric concentrations of CO_2_ and other greenhouse gas emissions, temperatures will increase and precipitation patterns will change [44]. Agriculture is arguably one of the sectors that are most sensitive and vulnerable to climate change. Wheat is considered the most significant cereal for human nutrition and is widely cultivated in many regions, such as Asia, Europe, and Northern Africa. Therefore, numerous studies have examined the impacts of climate change on wheat growth and yield in the major wheat-producing regions of the world [3, 4, 7, 27, 45–49].

In China, there are two types of wheat, winter wheat and spring wheat, which are named according to their growing period. Wheat is cultivated mainly in Hebei, Shanxi, Henan, Shandong, Anhui, Hubei, Jiangsu, Sichuan, and Shaanxi. Due to differences in climate conditions and precipitation patterns in different regions, the impacts of climate change/variability on wheat growth and yield differ as well. For example, You et al. [49] found that climate warming reduced wheat yield growth; a 1°C increase in temperature during the wheat growing period reduced wheat output by 3–10%. Similarly, Tao et al. [4] investigated climate-crop relationships and concluded that the total yield of wheat was estimated to change by -1.2*10^5^ t. Tao et al. [15] found that for the northern China, climate change increased wheat yield by 1.2–10.2%. In contrast, some studies have shown that the impact of climate change on the wheat yield was positive. Zhang et al. [37] suggested that in the past two decades, climate warming has benefited the wheat yield in the north-central region of China, although the effect of the average temperature and diurnal temperature range on the wheat yield was overall negative. Consistent with Zhang et al. [37] and based on experimental observations at agricultural meteorological stations in China, Tao et al. found that changes in temperature, precipitation and solar radiation from 1981 to 2009 jointly increased the wheat yield in northern China by 0.9–12.9% but reduced the wheat yield in southern China by 1.2–10.2% [15]. In addition, some researchers have investigated the future impacts of climate change on wheat yield. Tao et al. [14] evaluated the possible impacts of climate change on the winter wheat yield in the North China Plain under 10 climate scenarios and found a high probability that climate warming could increase winter wheat yield in the future. Simulated results have also indicated that relative to 1961–1990, the wheat yield will increase by up to 37.7% (18.6%), 67.8% (23.1%), and 87.2% (34.4%) with (without) CO_2_ fertilization effects during the 2020s, 2050s, and 2080s, respectively [14].

Additionally, agricultural technology progress is considered to be the main driving force for the increases in the wheat yield [28, 29]. Some complementary practices, including irrigation [29, 50–51], nitrogen fertilizer use [52–54] and management [51, 54], as well as the use of foliar fungicides [55], could mitigate wheat yield losses. Wang et al. [56] investigated the effects of irrigation and nitrogen regimes on wheat growth and yields based on field experiments in Henan Province and found that the wheat yield was significantly impacted by irrigation and nitrogen fertilization. You et al. [46] argued that using physical inputs could compensate for the negative effects of climate change on the wheat yield. Martín et al. [57] modeled the impact of climate change on the wheat yield under different climate change scenarios using a calibrated version of AFRCWHEAT2 and found that agriculture management factors such as altering sowing the date and the nitrogen fertilization increased wheat yields.

The abovementioned studies have not drawn consistent conclusions about the impact of climate change on wheat growth and yield due to the use of different time scale, geography locations and methods. In addition, these studies did not synthesize the effects of climate change and agriculture progress factors into crop yield-climate functions to examine their impact. Most importantly, agriculture technical progress, agriculture policy and cropping systems largely differ by province in China. Therefore, it is essential to investigate the impacts of climate change and agricultural technology on wheat at the provincial level. In this study, we used an ARDL model to simultaneously examine the effects of climate change and progress in agricultural technology on wheat yield in order to identify a conclusive relationship between them.

## Study area

**Fig 1:**
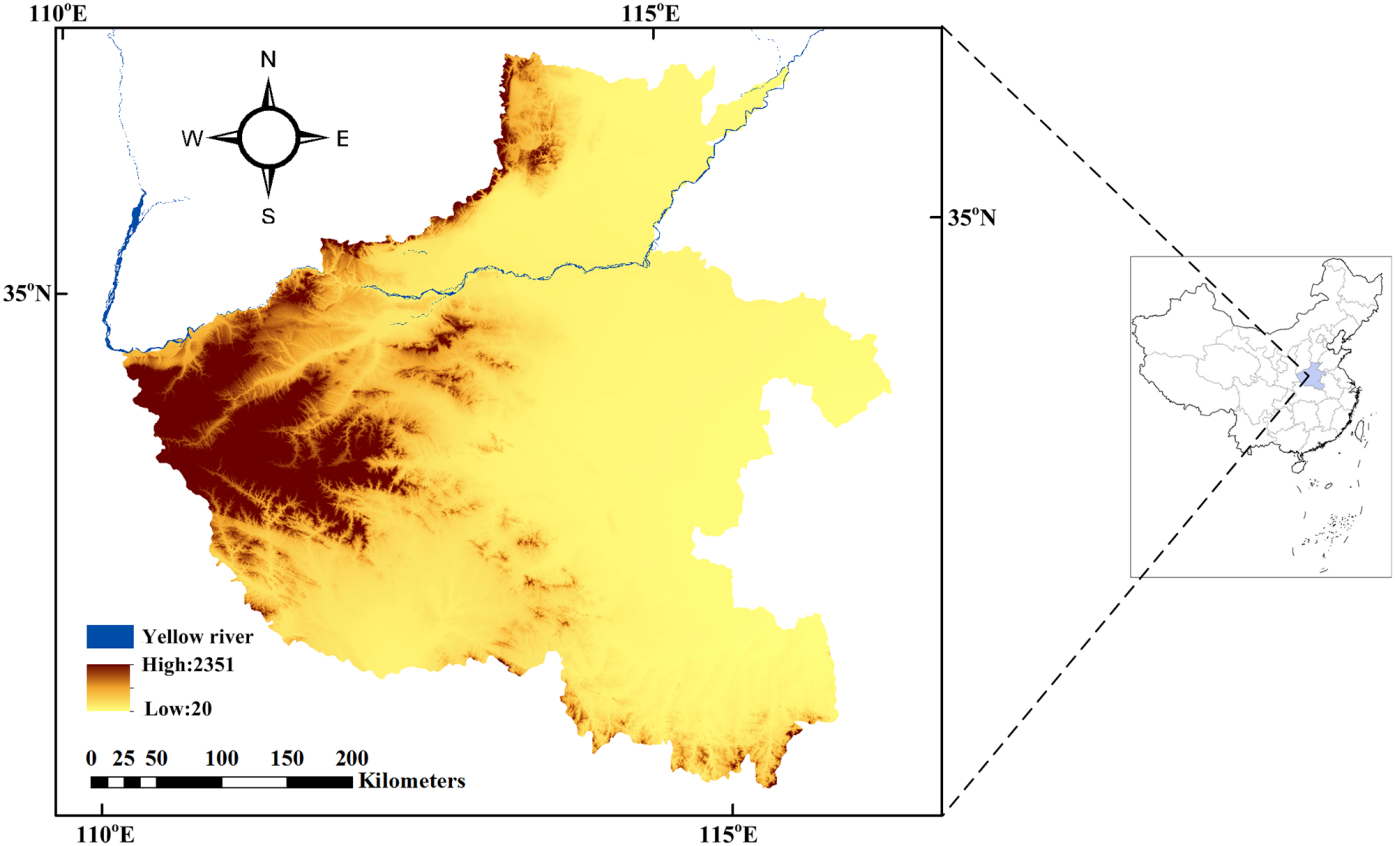
The location of the study area in China, including the main river (the Yellow River) and the elevation of the study area.

Henan Province is located in central China and covers an area of 167,000 km^2^. Henan has mountains on three sides and plains in the east and center (Fig 1). The areas of plains, mountains and hills in Henan account for 55.7%, 26.6%, 17.7% of the province, respectively. The elevation in Henan ranges from 20 m to 2351 m above sea level and decreases from west to east. The mountainous areas in Henan are located mainly in the west, north and south, respectively representing the Funiu, Taihang and Dabie Mountains. Henan Province is located in the climate transition zone from the subtropics to the warm temperate zone and is characterized by a humid/sub-humid climate of medium latitudes, with four distinct seasons. The annual frost-free period in the study area is approximately 180–240 days from north to south. The average annual temperature is 12–16°C, and the annual precipitation is 500–900 mm, 50% of which is concentrated in summer and is often accompanied by heavy rains. Agriculture is one of the most important industries in Henan Province. More importantly, Henan is one of most important bases of wheat cultivation, as it produces 25% of the wheat produced in China [38].

## Materials and methods

### Data collection and description

Agriculture couples natural reproduction and social reproduction. Therefore crop yields are jointly affected by natural factors (e.g., temperature, precipitation, soil, solar radiation) and socioeconomic factors (e.g., agriculture management, fertilizer use, and agriculture mechanism use). Previous studies indicate that climate change and agricultural technology are two important factors that influence crop yields [24, 28–29]. In this study, agricultural technology progress refers to the inputs of chemical fertilizers, planting areas, and mechanical progress. To examine the driving factors for increases in the wheat yield per unit area, we consider two types of factors. One type is the climate change factor, which includes temperature and precipitation. Temperature is the average temperature for entire wheat winter growth stage (from emergence to maturity, consisting of the period from last October to June). Similarly, precipitation is the total precipitation for entire wheat winter growth stage. The other type is the technical progress factor, which includes total power of agricultural machinery, fertilizer use, and the area used for wheat cultivation [28–31]. Additionally, the variable of area for wheat cultivation is selected to reflect the soil quality and regional government supports to wheat production.

Due to a lack of irrigation data for Henan Province, in this study and with reference to previous studies [14, 28–31], we considered six different variables to model the relationship between the wheat yield and the climate change and technological progress factors:

- M: total power of agricultural machinery in units of ten thousand kilowatts;
- F: aggregate quantity of fertilizer used in millions of tons;
- A: area for wheat cultivation measured in thousands of hectares;
- Y: wheat yield per unit area, defined as the wheat output divided by its cultivated area;
- P: total precipitation for the wheat growth period measured in millimeters; and
- T: average temperature in degrees Celsius for the wheat growth period.

**Fig 2:**
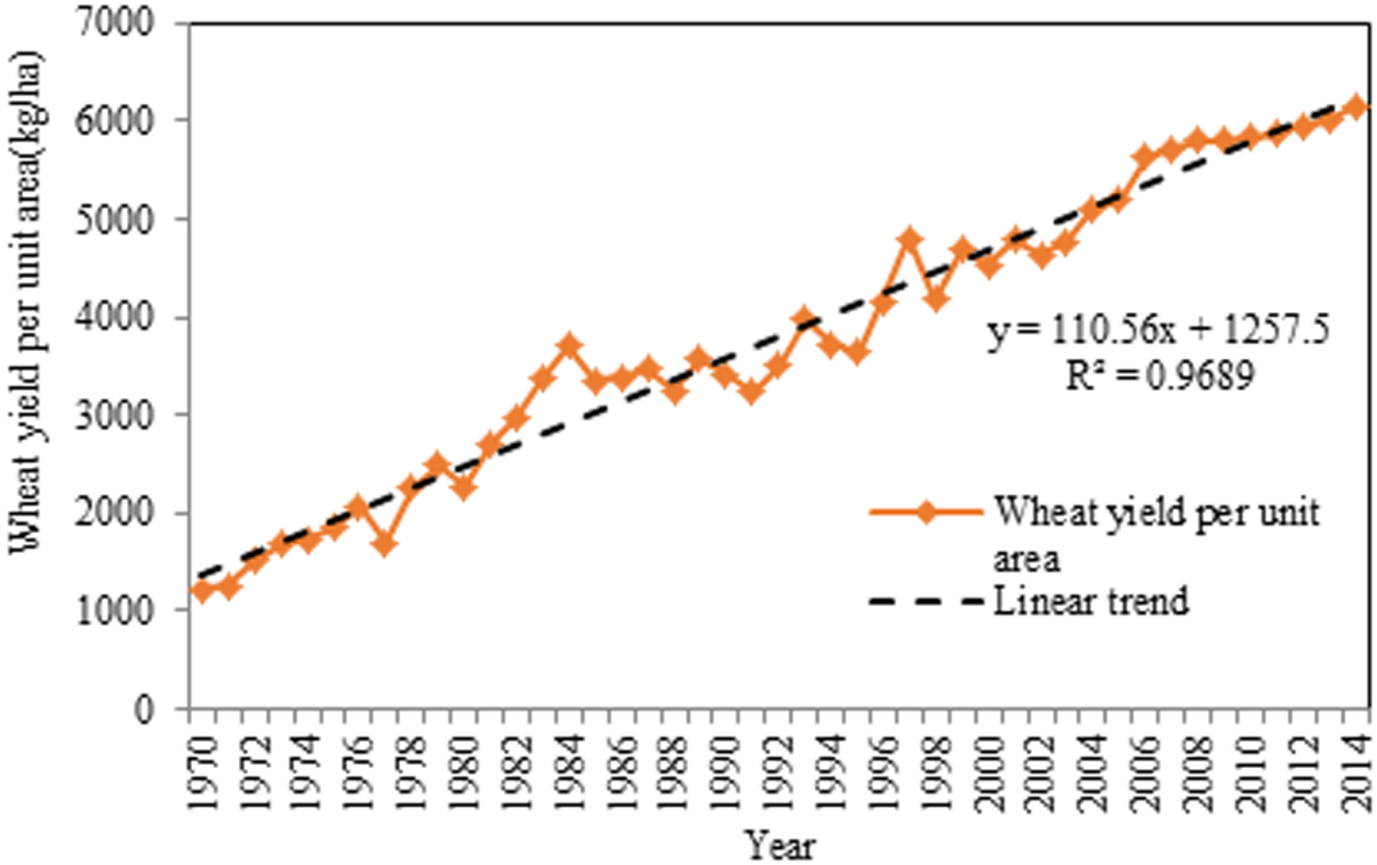
Annual wheat yield per unit area in Henan Province from 1970 to 2014. The orange line represents the annual wheat yield per unit area from 1970 to 2014. The black line represents line trend of the annual wheat yield per unit area from 1970 to 2014.

The wheat yield per unit area, or Y, was used as the dependent variable in the study’s model. P, T, M, F, and A were all included in the model as independent variables. This model used data from Henan Province spanning from 1970 to 2014. Precipitation and average temperature data were obtained from the China Meteorological Administration [58]. The other data (including M, F, Y and A) were collected from the National Bureau of Statistics of the People’s Republic of China [38]. Statistical profiles of the data are reported in Table 1. The wheat yield, the total power of agricultural machinery and fertilizer use showed large variations from 1970 to 2014. For example, the mean wheat yield per unit areais 3,800.31 kg/ha, while the wheat yield is as low as 1,226.39 kg/ha (minimum), and as high as 6,157.17 kg/ha (maximum). Fig 2 shows that the wheat yield per unit area during the wheat growth period sharply increased from 1970 to 2014(y = 110.56x+1257.5, R^2^ = 0.9689).

**Table 1:** Descriptive statistics for all the variables.

	Y	M	F	A	P	T
	(kg/ha)	(10^4^ kw)	(10^4^ t)	(10^3^ ha)	(mm)	(°C)
**Mean**	3800.31	4159.47	294.82	4,598.57	989.05	11.30
**Maximum**	6157.17	11,476.81	705.05	5,406.70	1286.62	12.60
**Minimum**	1226.39	189.60	15.40	3,639.90	854.13	10.25
**Std. Dev**.	1,475.15	3,620.20	228.54	561.75	91.72	0.55
**Skewness**	-0.05	0.73	0.38	-0.45	1.16	0.06
**Kurtosis**	1.91	2.07	1.79	1.90	4.45	2.41

**Fig 3:**
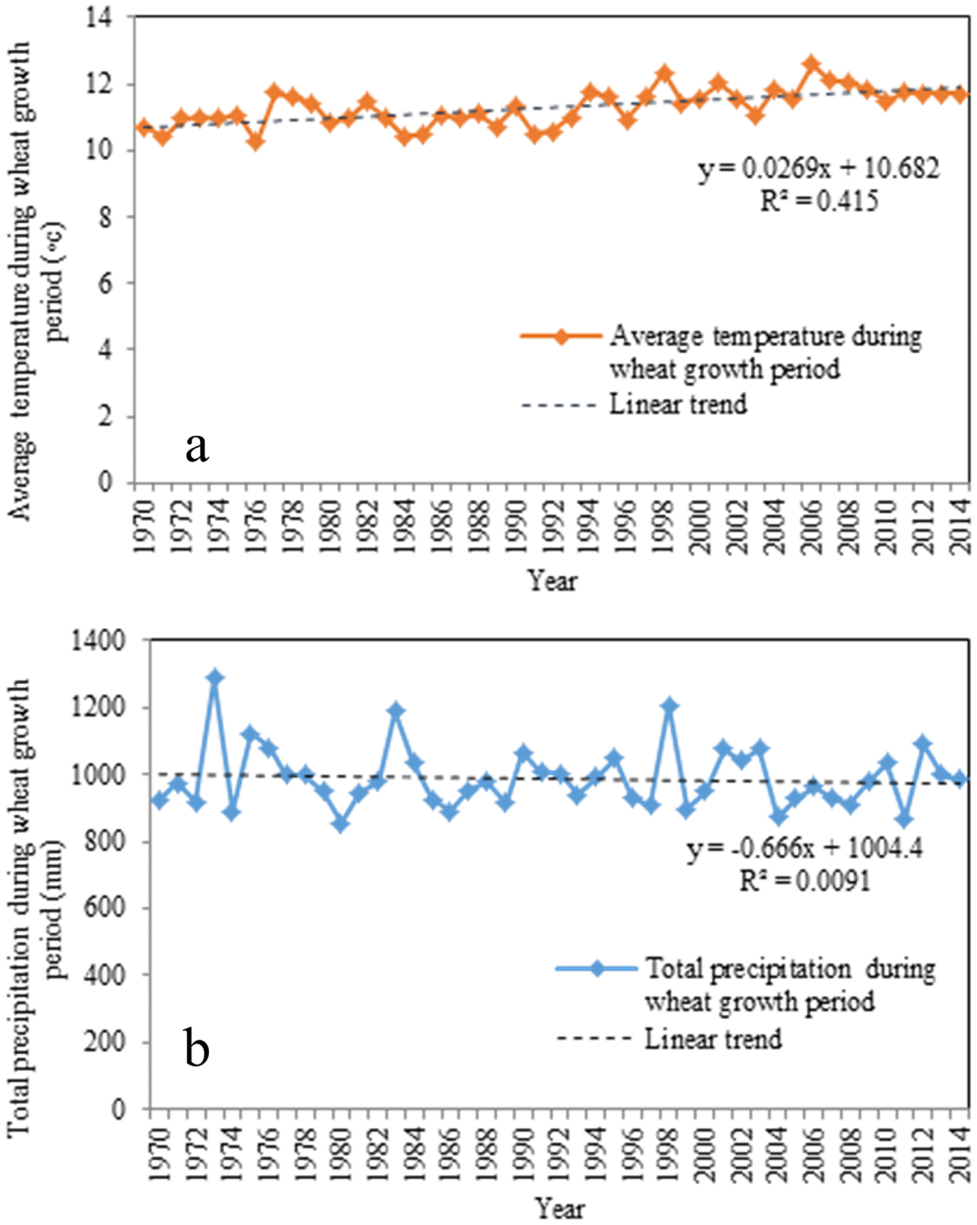
(a) Average temperature during the wheat growth period from 1970 to 2012 in Henan Province, China; (b) Total precipitation during the wheat growth period from 1970 to 2012 in Henan Province, China.

During the wheat growth stage, the maximum average temperature was 12.60°C, and the minimum average temperature was 10.25°C. Fig 3a shows that the average temperature during the wheat growth period gradually increased from 1970 to 2014(y = 0.0269x+10.682, R^2^ = 0.415). By comparison, the total precipitation fluctuated and showed a slight downward trend(y = 0.666x+1004.4, R^2^ = 0.0091) (Fig 3b). The total precipitation during the wheat growth period differed substantially by years. The maximum value for precipitation was 1286.62 mm in 1973, whereas the minimum value was 854.13 mm in 1980.

**Fig 4:**
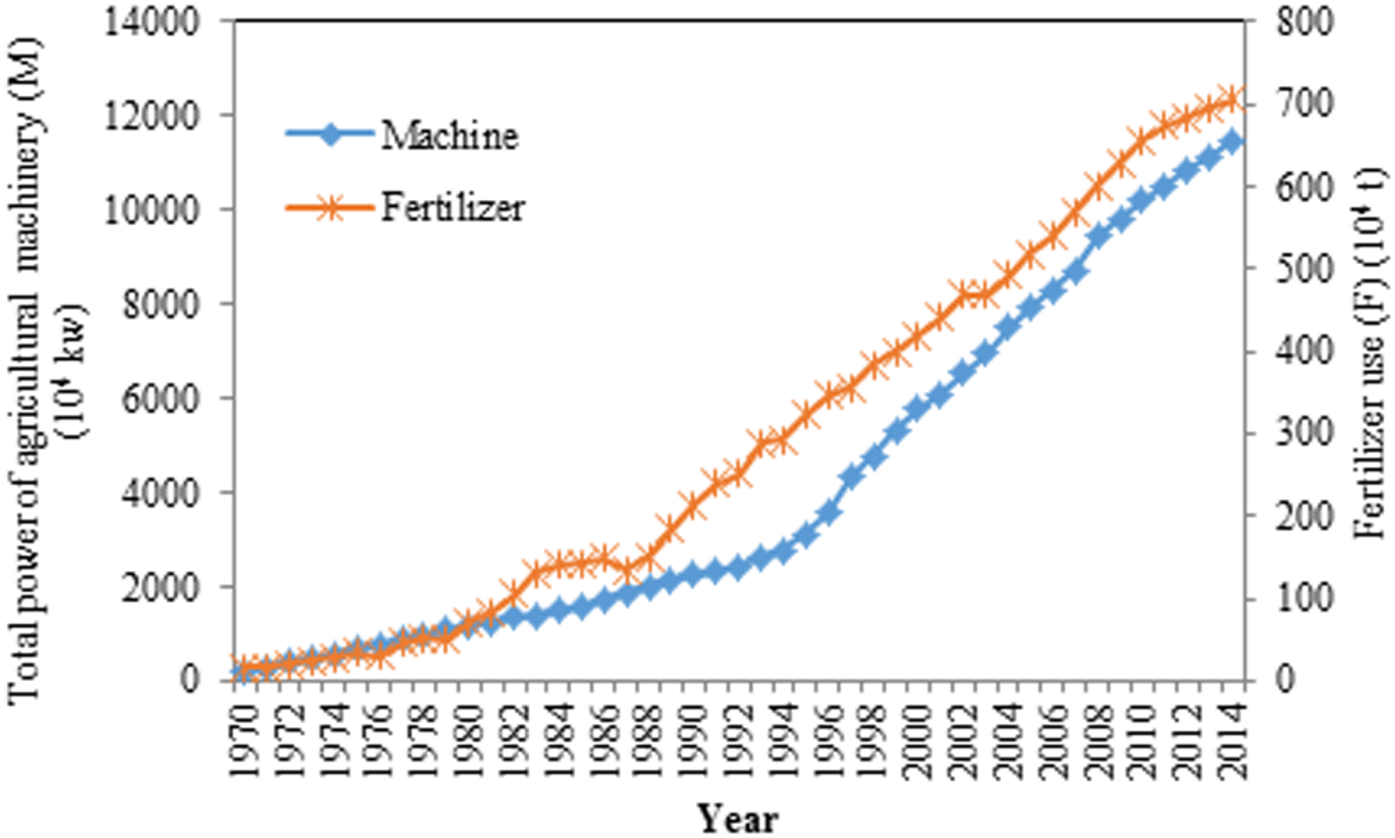
Annual total power of agricultural machinery and fertilizer use in Henan Province from 1970 to 2014. The orange line represents the amount of fertilizer use from 1970 to 2014. The blue line represents the total power of the agricultural machinery used from 1970 to 2014.

From Fig 4 and Table 1, we can see that the total power of agricultural machinery and fertilizer use increased from 1970 to 2014. The maximum value for the total power of agricultural machinery was 11,476.81 10^4^ kW in 2014, while the minimum value was 189.60 10^4^ kW in 1970. The largest value for fertilizer use was 705.05 10^4^ t in 2014. The lowest value appeared in 1970 and was 15.40 10^4^ t.

**Fig 5:**
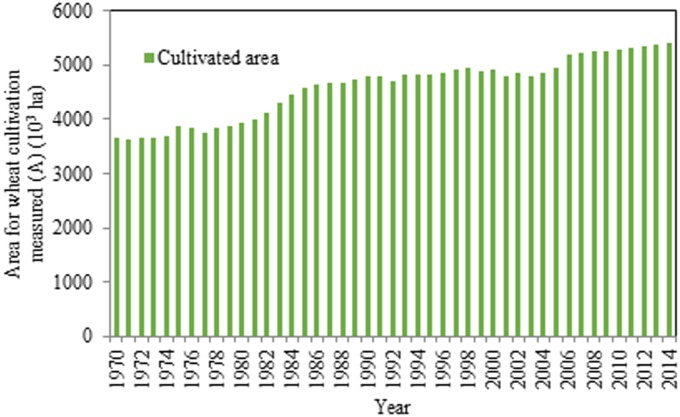
Annual area for wheat cultivation in Henan Province from 1970 to 2014.

Fig 5 and Table 1 together illustrate the changes in the area used for wheat cultivation. From 1970 to 2014, the area used for wheat cultivation gradually increased. It peaked at 5,406.70 10^3^ ha, while the minimum value was 3,639.90 10^3^ ha. The difference between the maximum and the minimum was not substantial, which indicates that the fluctuation was not significant.

### ARDL methodology

When time series data are employed in non-stationary or level form, a spurious regression problem may arise. To avoid a spurious regression, cointegration analysis was developed and employed to test the existence of a long-run relationship among time series variables [59]. The definition of cointegration is that, if two or more series are individually integrated, but some linear combination of them has a lower order of integration, then the series are said to be cointegrated [60]. In this study, to investigate the long-run relationship (which refers to two or more time series with a common drift way) among the variables being modeled, we adopted the ARDL bound test. In comparison to other cointegration techniques, the ARDL model has several advantages [40]: (1) ARDL can be applied irrespective of the same order of integration of the variables. Thus, ARDL is appropriate irrespective of whether the variables in the model are purely I(0) (which means that the variables were stationary in their level form and were integrated at order zero) or I(1) (which means that the variables were stationary in their differences and were integrated at order one) or mutually cointegrated. The order of integration, denoted I(d), is a summary statistic for a time series that reports the minimum number of differences required to obtain a covariance stationary series [61]. (2) ARDL can be used for small data samples. (3) ARDL can be applied to estimate short-run and long-run coefficients simultaneously. The short-run coefficients are the relationship between the deviation of the dependent variable from its long-run trend and the deviation of the independent variable from its long-run trend. More importantly, the ARDL methodology includes the bias-corrected bootstrap method and non-linear functions of the coefficients of the conditional error correction model, which can be used to estimate reliable statistical inferences for the long-run relationship among variables.

The relationship between the wheat yield and the independent variables was constructed as follows:

Y=f(M,F,A,P,T) (1)

We transformed all the variables into natural-log form to make Eq (1) estimable. The estimable form of the equation was modeled as follows:

$$\text{ln}\;Y=\beta_{1}+\beta_{2}\text{ln}\;M+\beta_{3}\text{ln}\;F+\beta_{4}\text{ln}\;A+\beta_{5}\text{ln}\;P+\beta_{6}\text{ln}\;T+\mu$$ (2)

In our study, we did not consider the different relationship among variables between different years, but focused on the short-run and long-run relationship between variables from 1970 to 2014. Therefore, the ARDL model does not include a year term. An ARDL representation of Eq (3) was formulated as follows:

$$\begin{array}{l} \Delta \text{ln}\;Y_{t}=\alpha_{0}+\sum_{i=0}^{p}\alpha_{1i}\Delta \text{ln}\;Y_{t-i}+\sum_{i=0}^{p}\alpha_{2i}\Delta \text{ln}\;M_{t-i}+\sum_{i=0}^{p}\alpha_{3i}\Delta \text{ln}\;F_{t-i}+\sum_{i=0}^{p}\alpha_{4i}\Delta \text{ln}\;A_{t-i}+\\ \sum_{i=0}^{p}\alpha_{5i}\Delta \text{ln}\;P_{t-i}+\sum_{i=0}^{p}\alpha_{6i}\Delta \text{ln}\;T_{t-i}+\beta_{11}\text{ln}\;Y_{t-1}+\beta_{12}\text{ln}\;M_{t-1}+\beta_{13}\text{ln}\;F_{t-1}+\beta_{14}\text{ln}\;A_{t-1}+\beta_{15}\text{ln}\;P_{t-1}\\ +\beta_{16}\text{ln}\;T_{t-1}+\varepsilon_{t} \end{array}$$ (3)

where *α*_0_is a drift component, *ε*_*t*_is a white noise term, and Δ is the first-difference operator. The ARDL approach estimated the (*p*+1)^n^ number of regressions to derive the optimal lag length for each variable. *p* is the maximum number of lags to be used. The terms with summation signs are the error correction dynamics. The coefficients *α*_1*i*_, *α*_2*i*_, *α*_3*i*_, *α*_4*i*_, *α*_5*i*_, *α*_6*i*_ represent the short-run dynamics of the model’s convergence to equilibrium. *β*_11_ − *β*_20_ are the long-run correlation coefficients, which indicate the long-run relationship among the variables.

To estimate the long-run relationship using the ARDL model for cointegration (used to judge the smooth or equilibrium relationship between multiple series of data), the first step is to estimate Eq (3) using ordinary least squares(OLS). Then, an F-test is performed to test whether there exists a long-run relationship among the variables. The F-statistic is a test of the hypothesis of no cointegration among the variables. The null hypothesis of no cointegration or no long-run relationship among the variables is set up as follows:

$$H_{0}:\beta_{11}=\beta_{12}=\beta_{13}=\beta_{14}=\beta_{15}=\beta_{16}=0$$

The alternative hypothesis suggests the existence of cointegration among the variables, which is as follows:

$$H_{1}:\beta_{11}\neq \beta_{12}\neq \beta_{13}\neq \beta_{14}\neq \beta_{15}\neq \beta_{16}\neq 0$$

The above hypotheses are judged using F-statistics of joint significance of *β*_11_ − *β*_20_, to test the existence of a long-run relationship among the variables.There are two critical values (Fl, Fu) for determining the bound test results [62–64]. Fu is the upper bound value. Fl is the lower critical bound value. If the F-statistic > Fu in the model, then the null hypothesis of no cointegration is rejected. This result would suggest the existence of a long-run relationship among the variables. However, if the computed F-statistic falls inside the critical value band, then an affirmative decision would not result. If F-statistic < Fl, then the inference of no cointegration among the variables could not be rejected.

The underlying assumptions for implementing the ARDL bound test were that all the variables must be integrated at the levels of I(0) or I(1) to allow for computation of F-statistics [19, 22]. Therefore, unit root tests are normally carried out on the variables that are introduced to the model [39]. The I(0) order represents the variables in the model that were stationary in their level forms, which indicats that the variables are integrated at order zero. The I(1) order indicates that the model variables are stationary in their first differences, which indicates that the variables are integrated at order one.

The lag orders of the variables are selected using Schawrtz Bayesian criteria(SBC) and Akaike’s information criteria(AIC). AIC selects the maximum relevant lag length. SBC selects the smallest possible lag length. The long-run relationship among the variables is estimated when the ARDL model is selected by the AIC or SBC criterion. Given the models for the data set in this study, lag orders were judged based on AIC.

Once a long-run relationship among the variables has been established, the error correction model(ECM) can be estimated. A general ECM of Eq (3) is formulated in Eq (4), which is as follows:

$$\begin{array}{l} \Delta \text{ln}\;Y_{i}=\alpha_{0}+\sum_{i=0}^{p}\alpha_{1i}\Delta \text{ln}\;Y_{t-i}+\sum_{i=0}^{p}\alpha_{2i}\Delta \text{ln}\;M_{t-i}+\sum_{i=0}^{p}\alpha_{3i}\Delta \text{ln}\;F_{t-i}+\sum_{i=0}^{p}\alpha_{4i}\Delta \text{ln}\;A_{t-i}+\\ \sum_{i=0}^{p}\alpha_{5i}\Delta \text{ln}\;P_{t-i}+\sum_{i=0}^{p}\alpha_{6i}\Delta \text{ln}\;T_{t-i}+\beta ECM_{t-1}+\varepsilon_{t} \end{array}$$ (4)

In fact, a last-period deviation from a long-run equilibrium would influence the short-run dynamics. In Eq (4), the coefficient of *ECM*_*t*−1_ suggests the speed of the adjustment by showing how quickly the variables return to the long-run equilibrium after a short-run shock.

To ensure the goodness of fit of the model, diagnostic and stability tests were conducted. Pesaran et al. [65] recommended using the Brown et al. [66] stability tests known as cumulative sum (CUSUM) and cumulative sum of squares(CUSUMSQ) to test the stability of the coefficient in the estimated models. If the plots of the CUSUM and CUSUMSQ statistics stay within the critical bonds of a 5% level of significance interval, the null hypothesis that all coefficients in the model are stable.

## Results

### Unit root test results

Therefore, we need to investigate the stationarity properties of the variables by employing the most commonly used unit root tests. To check the order of integration of each variable, we useed Augmented Dickey Fuller (ADF) and Phillips Perron (PP) unit root tests. Table 2 shows the results for the unit root test of the variables in the models. According to both the ADF and PP tests, the M, A, and Y variables were not stationary in their level forms but became stationary at their first differences at the 1% level of statistical significance. In addition, the variables F, P and T were both stationary in their level forms and at their first differences. In fact, the temperature and precipitation in Henan Province are impacted by other provinces’ temperature and precipitation levels due to the space-adjacent relationship. However, in this study, we only considered the time-series relationship among the variables based on historical data; thus, we did not run spatial stationary tests of temperature and precipitation in Henan Province.

**Table 2:** Unit root test results.

	ADF test statistics		PP test statistics	
variables	level	1^st^ Differences	levels	1^st^ Differences
**lnM**	-2.0620	-5.9160***	-3.9435***	-6.1087***
**lnF**	-3.0691**	-6.7728***	-3.9720***	-6.8467***
**lnA**	-1.1478	-5.5781***	-1.1211	-5.5937***
**lnY**	-2.1996	-8.6963***	-2.7935*	-8.8649***
**lnP**	-7.8215***	-8.0887***	-7.9233***	-30.1145***
**lnT**	-3.6148***	-7.3879***	-3.6148***	-19.9617***

***Significance at 1% level;

** Significance at 5% level;

* Significance at 10% level

### ARDL bound test for cointegration analysis

To test the long-run and short-run relationships among the variables, it was first necessary to determine whether cointegration relationships existed among the variables. Table 3 shows the results for cointegration from applying the ARDL bound test. The computed F-statistic of 4.306 is higher than the upper-bound critical value (at the 2.5% significance level) when the wheat yield per unit area is the dependent variable. Therefore, these results indicated the existence of cointegrating relationships among the variables when the wheat yield per unit area is used as the dependent variable.

**Table 3:** ARDL bound test for cointegration.

F-Statistic	Critical Value	Lower Bound Value	Upper Bound Value	Conclusion
4.306	1%	3.41	4.68	Cointegration
	2.5%	2.96	4.18	
	5%	2.62	3.79	
	10%	2.26	3.35	

The lag lengths applied in testing the model were selected based on Akaike’s information criteria(AIC) and resulted in ARDL (1,1,2,1,2,1) models. The calculated F-statistics of the bound tests are compared with the critical values obtained from Persaran [39] for k = 6.

### Parameter estimation and interpretation

Because the existence of cointegration among the variables was confirmed, the ARDL bound test for the short-run and long-run relationships between wheat yield per unit area and the factors of climate change and technical progress was estimated. Table 4 summarizes the results of Eqs (3) and (4), which are based on the sample period between 1970 and 2014.

**Table 4:** Results of short-run and long-run coefficients from ARDL(1,1,2,2,2,1) model.

Dependent variable is *ln*Y				
45 observations from 1970 to 2014 were used for the estimation				
	Variable	Coefficient	Standard error	t-Ratio[Prob]
**Long-run**	lnM	0.2143***	0.0664	3.23[0.003]
**coefficients**	lnF	0.1969**	0.0789	2.42[0.011]
	lnA	-0.0384	0.4685	-0.08[0.935]
	lnP	-0.5536*	0.2890	-1.92[0.066]
	lnT	-0.6976	0.5071	-1.38[0.180]
**Short-run**	ΔlnM	0.1974	0.2427	0.81[0.423]
**coefficients**	ΔlnF	-0.2505**	0.1229	-2.22[0.035]
	ΔlnA	1.9851***	0.5950	3.34[0.002]
	ΔlnP	0.3071*	0.1689	1.82[0.080]
	ΔlnT	0.1608	0.2692	0.60[0.555]
**Error correction coefficient**	ECM_t-1_	-0.8167***	0.1639	-4.98[0.000]
	Cons	3.9876**	1.6782	2.38[0.025]
	Log likelihood = 111.8696 R-squared = 0.7961 VIF = 34.30		Adj R-squared = 0.6942 Root MSE = 0.0222	

***Significance at 1% level;

** Significance at 5% level;

* Significance at 10% level

The long-run coefficients of agricultural machinery and fertilizer use are statistically significant (Table 4). These results suggest that a 1% increase in machine use leads to a 0.21% (Table 4, row 4, column 3) increase in the per unit area wheat yield. Furthermore, the magnitude of 0.1969 implies that a 1% increase in fertilizer use will increase the per unit area wheat yield by 0.19% (Table 4, row 5, column 3) when long-run effects are considered. In the long term, the area used for wheat cultivation does not significantly impact the wheat yield per unit area. It is worth noting that the long-run coefficient of precipitation is negative, which implies that an increase in precipitation leads to a decreased wheat yield per unit area. Indeed, a 1% increase in precipitation leads to a 0.55% (Table 4, row 7, column 3) decrease in the wheat yield per unit area. However, the impact of temperature on the wheat yield per unit area in the long run is found to be not significant during the whole wheat growth period.

For the short-run relationship, the coefficient of machine use is not significant when the wheat yield per unit area is the dependent variable. The coefficient of fertilizer use is negative. The coefficient of the wheat cultivation area shows that there is a positive and significant relationship between the area used for wheat cultivation and the wheat yield per unit area. This result implies that in the short run, an increase in the wheat cultivation area will play an important role in increasing the wheat yield in Henan. A 1% increase in the wheat cultivation area leads to an increase in the wheat yield per unit area by 1.98% (Table 4, row 11, column 3). Conversely, in the short run, precipitation has a significantly positive impact on the wheat yield. The sign of the precipitation coefficient is positive and statistically significant in the short run, which is the opposite of the relationship between precipitation and the wheat yield in the long run. The coefficient of P is 0.3071 which implies that a 1% increase in precipitation will lead to a 0.31% (Table 4, row 12, column 3) increase in the wheat yield per unit area.

The lagged error correction coefficient, ECM_t-1_, is correct in sign, and significant in verifying the established cointegration relationship among the variables. The coefficient of ECM_t-1_ shows the speed of the adjustment back to the long run equilibrium after a short run shock. In Table 4, the coefficient of ECM_t-1_ is -0.8167 (Table 4, row 14, column 3), which is between 0 and -1 and means that approximately 0.82% of the disequilibria in the wheat yield per unit area of the previous year’s shock adjust back to the long-run equilibrium in the current year.

We can infer from the results of the analysis that promoting the efficiency of the agricultural machinery used would be a good way to increase the wheat yield per unit area in the long run. In addition, appropriately increasing the aggregate quantity of fertilizer used may also be worthy of consideration in the long run. Important conclusions can be drawn from the short-run test results. To resolve any the adverse effects on the wheat yield, it may be necessary to pay more attention to expanding the area of wheat cultivation. It should be noted, however, that it might not be possible to immediately expand the land area for wheat cultivation because the available arable land could be limited. In fact, mountainous and hilly areas account for 44.3% of Henan Province, which makes cultivating more arable land improbable. Converting barren land in mountainous and hilly areas to arable land for the cultivation of wheat is a laborious task that takes substantial time. Thus, the best remedy in response to the reduction in the wheat yield may be to better utilize the arable land by increasing the cultivation intensity through the use of agricultural machinery.

### Stability test

**Fig 6:**
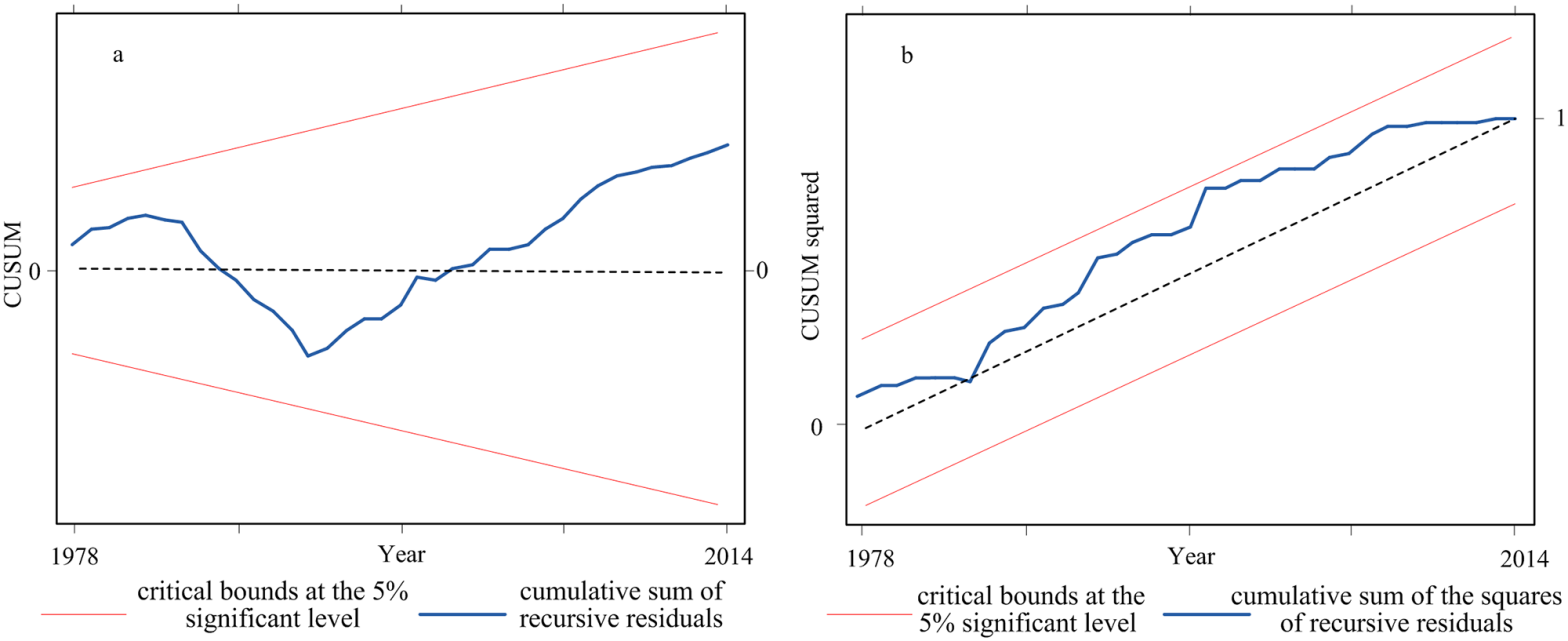
(a) Plot of the cumulative sum of the recursive residuals; (b) Plot of the cumulative sum of squares of the recursive residuals. The straight red lines represent the critical bounds at the 5% significant level. The blue lines show the statistics for the cumulative sum of the recursive residuals and the cumulative sum of the squares of the recursive residuals.

For the goodness of fit for the ARDL model, tests using the cumulative sum (CUSUM) and the CUSUM of squares (CUSUMSQ) were carried out after confirming the cointegration relationship among variables. CUSUM and CUSUMSQ tests were conducted based on the recursive regression residuals as suggested by Brown [66]. If the plots of the statistics fell inside the critical bounds at the 5% significance level, it would suggest that the calculated results of the coefficients of the ARDL model were stable. The model stability are shown in Fig 6; they suggest that the goodness of fit of the ARDL model was good.

## Discussion

The ARDL regression indicated that in the long run, the relationship between the average temperature and the winter wheat yield is not significant, which was inconsistent with previous studies exemplified by [15] who, used data from agricultural meteorological stations to find that the winter wheat yield was positively correlated with the temperature in Henan Province. However, Zhang and Huang [37] found that the positive effect of temperature on the wheat yield only existed in the southern part of Henan Province, while in northeast, the effect was negative. In fact, the average temperature during wheat growing season from 1970 to 2014 showed a slightly rising tendency, but it did not appear obviously fluctuation and was around at 11°C (Fig 3a) which was well within the physiological tolerances of the local wheat varieties. Therefore, it was not surprised that there was not clear impact of temperature on wheat yield. In order to ensure winter wheat growing, it needs accumulated temperature of 2100–2400°C during wheat growth period. In a follow-up study, the relationship between wheat yield and accumulated temperature should be tested. In addition, our result also showed that the precipitation has a negative impact on the wheat yield in Henan province, which is consistent with Li et al. [67], who used county-level wheat planting area and production data. This may be related to the fact that Henan province is dominated by non-rain-fed agriculture. Excessive rainfall will be harm to crop growth. Li et al. [67] argued that precipitation has a higher association with the wheat yield at smaller scales (0.5°, 2°/2.5°) than at larger scales (4°/5°); nevertheless, at all levels of aggregation, the wheat yield has a strong association with temperature. Thus, the correlation between the wheat yield and climate was not consistent. These differences may be due to different spatial scales or geographic location, such that a strong correlation may be found at one scale or in one place, but such a result cannot be assumed to explain other scales or other regions [67, 68]. Therefore, in the future study, the relationship between climate factor and crop yield should be examined at different spatial scale.

Our results demonstrated that the increased wheat yield per unit area in Henan Province is mainly explained by agriculture technical progress, such as increasing the input of machines and fertilizers, which is consistent with previous research [28, 29] [57]. Additionally, effective irrigation was an important factor in ensuring wheat growth, especially in non-rain-fed regions [51, 69]. Liu et al. [69] argued that the amount of irrigation water applied is very important to quantify the contribution of irrigation to the wheat yield. Wang [70] examined the share of effective irrigation area of the total cultivated area to test its impact on wheat yield. However, in this study, we found that there was a strong correlation between the effective irrigation area and the wheat planting area (r = 0.82) [58], as Henan is dominated by non- rain-fed agriculture. Furthermore, due to a lack of consumption data for irrigation water, we did not investigate this factor. In addition to increasing the use of physical inputs, R&D investment and institutional changes (e.g., the household contract responsibility system with remuneration linked to output) in China are also considered to be stimulating factors that improve the wheat yield [46], which should be investigated in a follow-up study.

Fertilizer use was considered as a dominant factor in the increase of wheat yields, especially in the eastern production areas of the North China Plain [71].Our results once again demonstrated this conclusion. However, in our study, we found that the effect of fertilizer use is negative when short-run effect is considered. The reason may be that, in the short term, the use of excessive fertilizer will destroy soil structure and break the soil ecosystem balance, resulting in wheat production. Since 1980s, even though the average yield of cereal crops is stagnating, fertilizer application has continued to increase in China, which led to decrease fertilizer use efficiency and increase environment pollution [71–73]. Tong et al. [72] argued that the higher the fertilizer input, the lower the yield per unit chemical fertilizer input. Thereby, in the future study, the relationship between fertilizers input intensity and wheat yield per unit area should be investigated.

In fact, the wheat yield is jointly influenced by climate factors, the use of modern inputs and crop management, and it is difficult to separate one factor’s influence on the wheat yield from that of the other factors [49]. Most previous studies failed to employ climate factors, agriculture technical progress factors (e.g., fertilizer, machinery, irrigation) and agriculture management practices in the same crop yield-climate functions. Therefore, it is necessary to build a unified framework or method to examine the relationship between the wheat yield, climate change and agricultural technology progress. You et al. [49] tested the effects of physic input (e.g., seeds, fertilizer, pesticide, machinery and so on) and climate factors (e.g., temperature, rainfall and solar radiation) on wheat yield using Cobb-Douglas function. However, when doing so, if a co-integration relationship between non-stationary time series data does not exist, then the regression models based on the time series data are likely to produce spurious regression results. The ARDL model can effectively avoid the spurious regression problem. Additionally, it can not only consider the long-run equilibrium relationship and the short-run fluctuation relationship between variables but also can effectively distinguish between them [74]. Thus, the ARDL model is an effective method for estimating the relationship between climate change, technical progress and the wheat yield.

Most of the previous studies have focused on investigating the impact of climate change on crop yields at the national level [44, 46] or the regional level [37]. However, in China, the climate conditions, crop varieties and management patterns vary from each province. Hence, in order to design well-targeted adaptation policies and to ensure food security, there is a need to extend this study to other provinces. Such follow-up studies should not only focus on the impact of climate factors, agriculture technology progress and other input factors on crop yields, but also pay more attention to the differences among the different provinces.

## Conclusions and implications

In this paper, we examined the relationships among the wheat yield per unit area, machine use, the area used for wheat cultivation, the aggregate quantity of fertilizer used, precipitation and temperature in Henan Province over the period between 1970 and 2014. Based on the analytical results of this work, two issues can be resolved. One is whether the threat of climate change impacted the wheat yield per unit area in Henan Province. The other is whether technical progress, i.e., machine use and aggregate quantity of fertilizer used, increased the wheat yield per unit area. For this purpose, we applied the ARDL model to test the influence of climate change and technical factors on the wheat yield per unit area in the long run. The results of the ARDL bound test for cointegration suggest that there is cointegration among the variables.

In the long run, the results of the analysis show that the coefficients of machine use and the aggregate quantity of fertilizer used were positive and statistically significant, suggesting that these factors have improved the wheat yield per unit area in Henan Province. In contrast, the results also show that precipitation during the wheat growth period has led to a decrease in the wheat yield per unit area. In the short run, the coefficient of the aggregate quantity of fertilizer used was negative. Precipitation and the area used for wheat cultivation had a significantly positive impact on the wheat yield. There was no significant short-run or long-run impact of temperature on wheat yield per unit area in Henan Province. It is concluded that wheat yield per unit area are mainly improved by machine use and aggregate quantity of fertilizer used from 1970 to 2014 in Henna province.

The findings of this study have several policy implications that could ensure continuous increases in the wheat yield per unit area and food security under climate change in Henan Province. Large-scale mechanical operations and intensive cultivation could be effective measures to increase the wheat yield. Therefore, central and local governments should fund agriculture mechanical operations. The amount of cultivated area was not the main driving factor for the increased wheat yield in the long term. Protecting the arable land cultivated for wheat is necessary to increase the wheat yield because the amount of arable land is limited. Precipitation had a negative impact on the wheat yield. The water required for wheat growth in Henan Province is mainly derived from irrigation. Therefore, under climate change, the government may also need to repair and expand the existing irrigation facilities to avoid the negative effects of drought. In addition, the present study also found evidence that fertilizer use positively impacts the wheat yield per unit area in the long run. The uptake of nitrogen, phosphorus and potassium during the period of wheat growth must change based on the characteristics of particular wheat varieties, cultivation techniques, the soil, and climate change. In addition, the required amount and proportion of nutrient absorption is different during each wheat growth period. Thus, the government may need to make fertilizer available to farmers at no cost or at subsidized prices. In addition, agro-technicians could be arranged to guide farmers regarding the reasonable use of fertilizers, based on the climate, the soil, wheat variety and growth period characteristics.

## Supporting information

S1 Table Historical statistic data for all variables in Henan Province from 1970 to 2014. (XLSX)
